# Supplementary material for: IL‐2/IL‐7‐inducible factors pioneer the path to T cell differentiation in advance of lineage‐defining factors
Source: EMBO J. 2020 Sep 15;39(22):e105220. doi: 10.15252/embj.2020105220 (PMC7667885; doi:10.15252/embj.2020105220)
Supplement: Supplementary file 1 — Expanded View Figures PDF [file EMBJ-39-e105220-s001.pdf]

Expanded View Figures

Figure EV1. A subset of pDHSs in previously activated T cells are dependent on IL-2.

A *Il2ra* mRNA levels in T_B IL-2 and T_B IL-2nil. Standard deviation is shown from 3 replicates. *P*-values were calculated from the RNA-Seq data using Limma with Benjamini-Hochberg correction for multiple testing.

B Flow cytometry plots showing the levels of Annexin V and Propidium Iodide (PI) staining on T_B IL-2 and T_B IL-2nil cells following purification.

C DNase-Seq tag density plots showing all peaks for replicate 1 (left), using panels taken from Fig 2A, and replicate 2 (right) ordered individually according to fold change of T_B IL-2/T_B IL-2nil. The number of peaks which are twofold enriched in T_B IL-2 compared to T_B IL-2nil and are common to both replicates is shown below.

D Venn diagram showing the overlap between the 1,000 IL-2 pDHSs and the 2,882 pDHSs defined in *in vivo*-derived CD4 memory T cells (Bevington *et al*, 2016).

E Average DNase-Seq tag density profile at the 2,882 memory pDHSs and 2,882 control invariant DHSs in T_B IL-2 and T_B IL-2nil.

F DNase-Seq tag density plots showing all peaks detected in T_B IL-2 DMSO and T_B IL-2 Ruxolitinib (33,621) ordered by increasing fold change of tag count for T_B DMSO compared to T_B Ruxolitinib.

G Average DNase-Seq tag density profile at the at the 1,000 IL-2 pDHSs in T_B IL-2 DMSO and T_B IL-2 Ruxolitinib.

H, I UCSC genome browser tracks for the *Il17a* (J) and control regions at the CD3 gene cluster and *Tbp* locus (K). The blue box indicates a DHS which is inhibited by Ruxolitinib.

 © 2020 The Authors

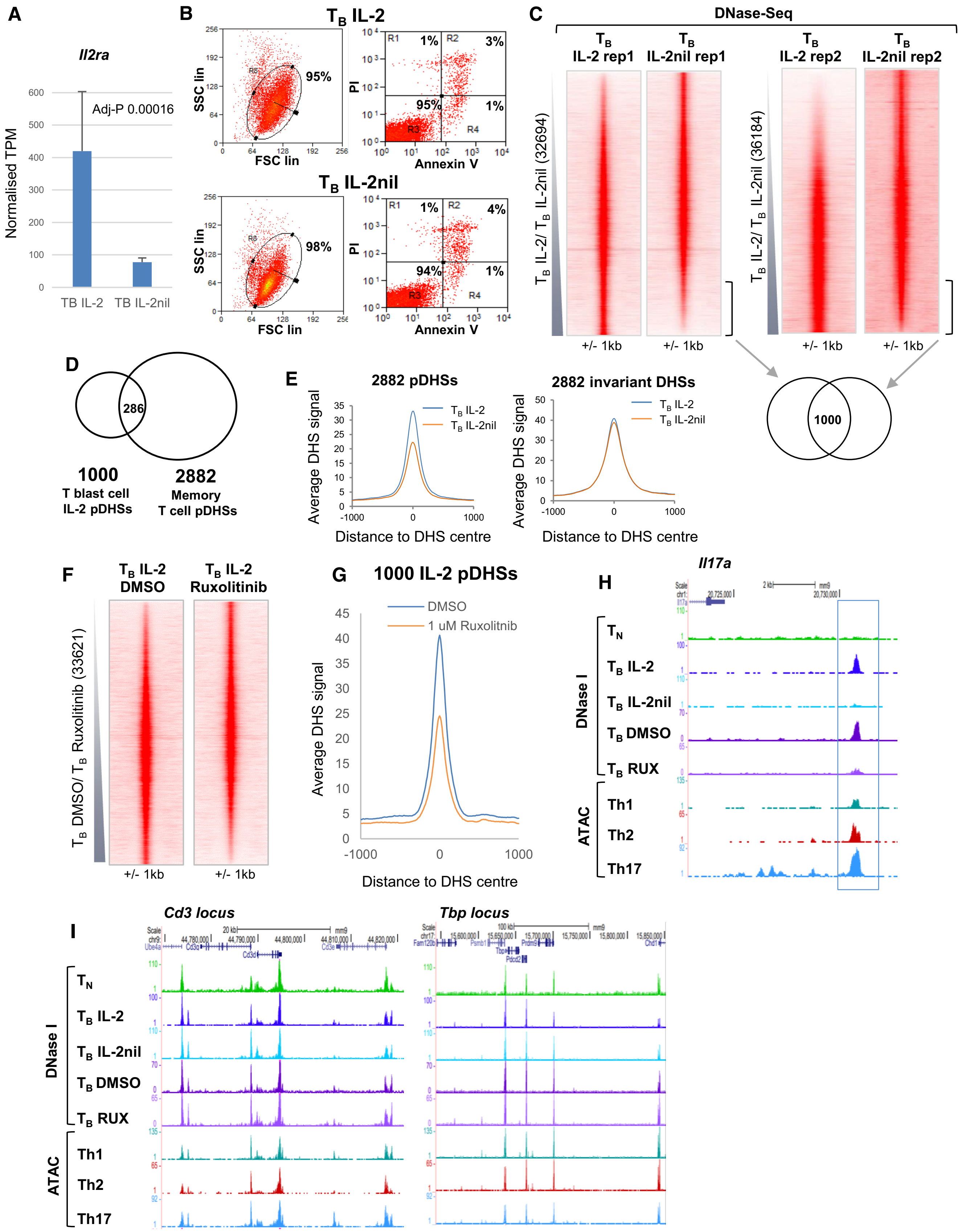
A
Il2ra
Normalised TPM
Adj-P 0.00016
TB IL-2
TB IL-2nil
B
TB IL-2
TB IL-2nil
SSC lin
FSC lin
PI
Annexin V
95%
98%
C
DNase-Seq
TB IL-2 rep1
TB IL-2nil rep1
TB IL-2 rep2
TB IL-2nil rep2
TB IL-2/ TB IL-2nil (32694)
TB IL-2/ TB IL-2nil (36184)
+/- 1kb
1000
D
286
1000
T blast cell IL-2 pDHSs
2882
Memory T cell pDHSs
E
2882 pDHSs
2882 invariant DHSs
Average DHS signal
Distance to DHS centre
TB IL-2
TB IL-2nil
F
TB IL-2 DMSO
TB IL-2 Ruxolitinib
TB DMSO/ TB Ruxolitinib (33621)
+/- 1kb
G
1000 IL-2 pDHSs
DMSO
1 uM Ruxolitinib
Average DHS signal
Distance to DHS centre
H
Il17a
DNase I
ATAC
TN
TB IL-2
TB IL-2nil
TB DMSO
TB RUX
Th1
Th2
Th17
I
Cd3 locus
Tbp locus
DNase I
ATAC
TN
TB IL-2
TB IL-2nil
TB DMSO
TB RUX
Th1
Th2
Th17

Figure EV1.

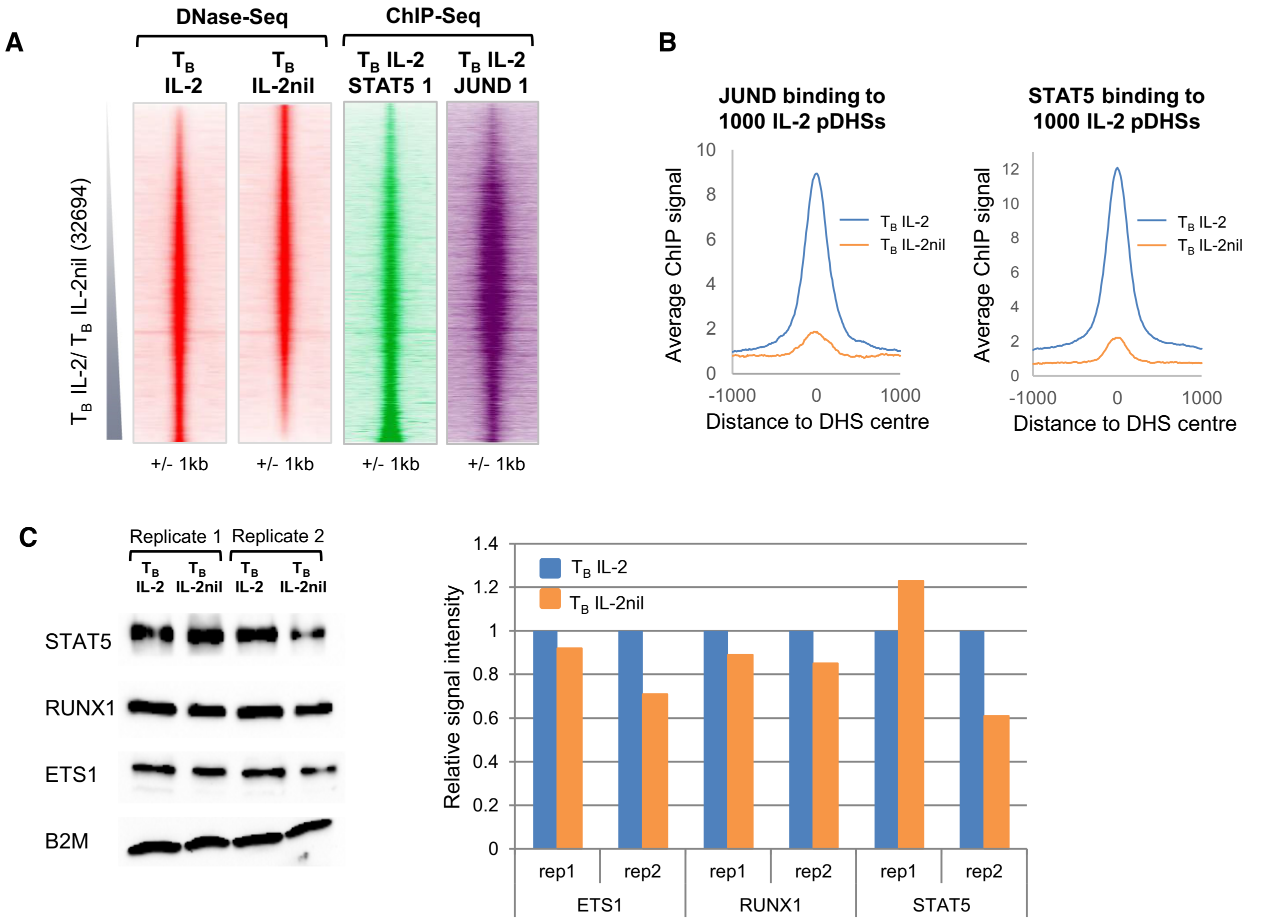

Figure EV2. IL-2 pDHSs bind IL-2 regulated factors.

A DNase-Seq tag density plots, using T_B IL-2 and T_B IL-2nil DNase-Seq data from Fig 2A, showing ChIP samples for a second replicate of T_B IL-2 STAT5 and T_B IL-2 JUND, and ordered according to the fold enrichment of DNase-Seq tag counts in T_B IL-2 compared to T_B IL-2nil.

B Average ChIP-Seq signal at the 1,000 IL-2 pDHSs in T_B IL-2 and T_B IL-2nil for JUND and STAT5.

C Western blot analyses showing protein levels of RUNX1, ETS1, and STAT5 in T_B IL-2 and T_B IL-2nil. Signals were quantitated and the value for T_B IL-2 set to 1.

 © 2020 The Authors

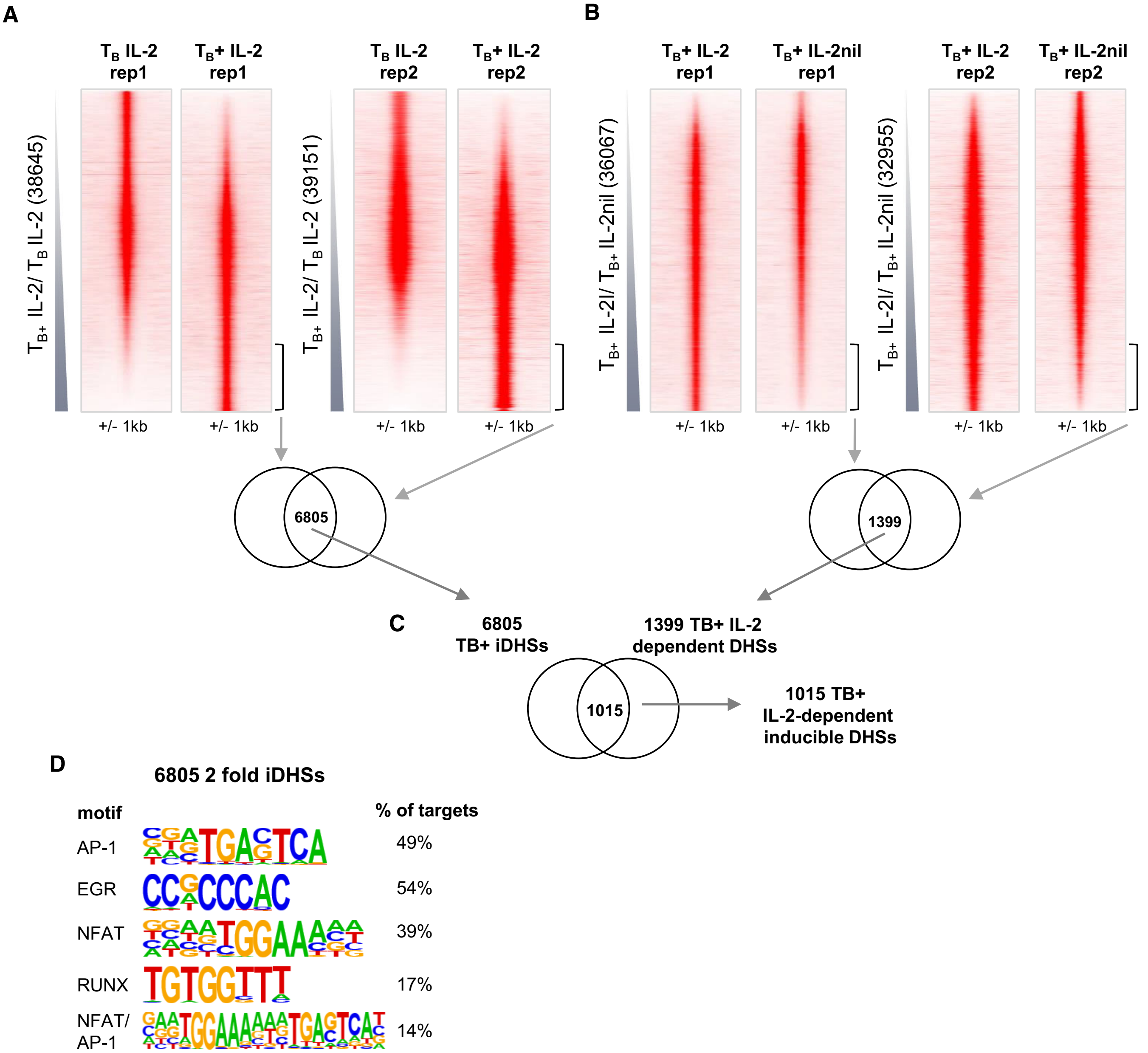

Figure EV3. IL-2 signaling is required for inducible DHS formation.

A, B DNase-Seq tag density plots showing all peaks detected in T_B IL-2 and T_B+ IL-2 (A) and T_B+ IL-2 and T_B+ IL-2nil for the two replicate samples (B). The numbers of peaks which are twofold enriched and are common to both replicates are shown below.

C Identification of 1,015 IL-2-dependent inducible DHSs as the overlap between the 6,805 iDHSs and the 1,399 IL-2-dependent DHSs.

D HOMER *de novo* motif analysis of the 6,805 T_B+ IL-2 iDHSs.

© 2020 The Authors

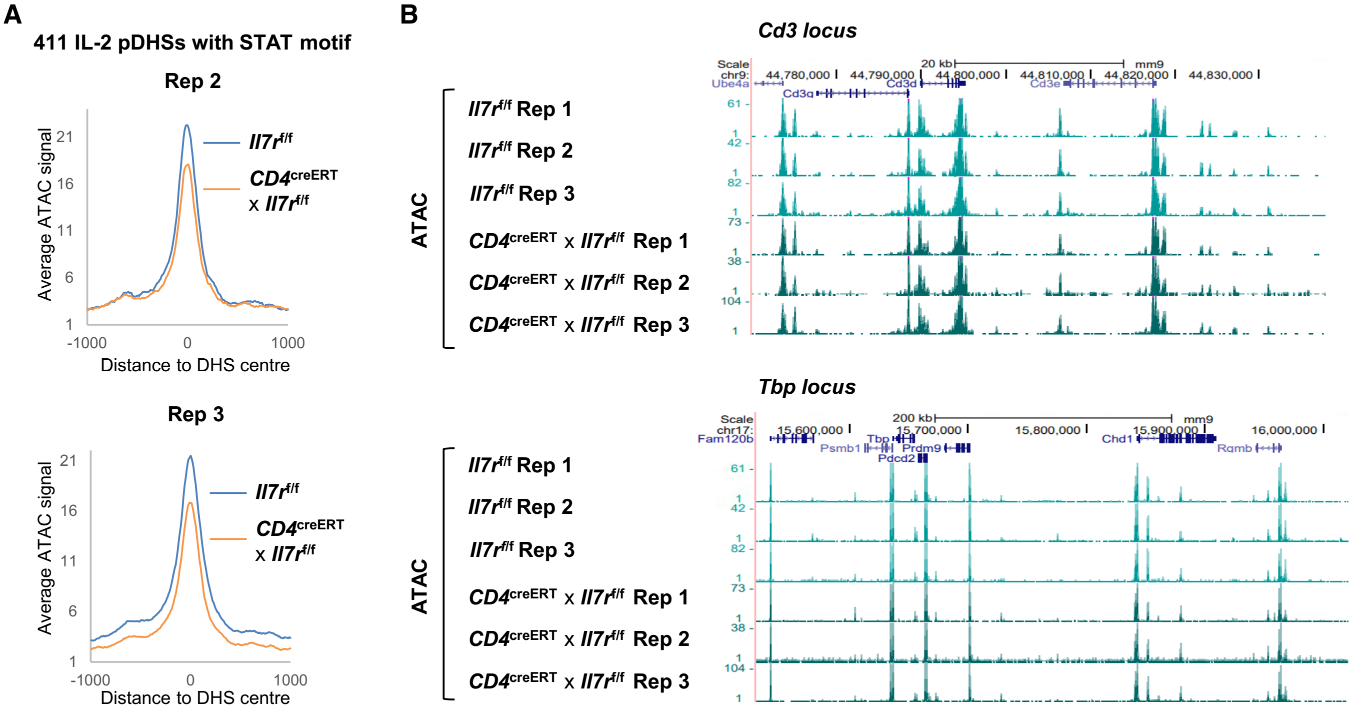

Figure EV4. IL-2 pDHSs are reduced in antigen-specific memory T cells upon deletion the IL-7 receptor.

A Average ATAC signal at the 411 IL-2 pDHSs which contain a STAT-binding motif in *CD4*^{creERT2} × *Il7r*$^{f/f}$ and *Il7r*$^{f/f}$ for replicates 2 and 3.

B UCSC genome browser tracks showing control regions at the CD3 gene cluster and a 500 kb region of the *Tbp* locus for the replicate ATAC-Seq samples of *CD4*^{creERT2} × *Il7r*$^{f/f}$ memory T cells and *Il7r*$^{f/f}$ control memory T cells.

 © 2020 The Authors
